# Nonopioid Drugs for Postoperative Pain: A Selection Governed by Choices of the Authors of Academic Articles

**DOI:** 10.2174/0127724328327509240919102209

**Published:** 2024-10-01

**Authors:** Igor Kissin, Kamen V. Vlassakov

**Affiliations:** 1 Department of Anesthesiology, Perioperative and Pain Medicine, Brigham and Women’s Hospital, Harvard Medical School, Boston, Massachusetts, 75 Francis Street, MA02115, USA

**Keywords:** Nonopioid, postoperative pain, academic articles, opioid crisis, nonopioid analgesics, academic articles

## Abstract

The opioid crisis has profoundly changed the interest in using nonopioid analgesics. This review identified nonopioid drugs receiving the most interest in the treatment of postoperative pain. Publication-based interest, which reflects the authors’ choices of subjects for academic articles, was used to show the shifts in their interest. The authors’ choices of a particular drug for an article’s subject were regarded as reflective of the collective opinion of experts most knowledgeable on the subject. The frequency with which a drug was the topic of an article was measured with the use of specific bibliometric indices. They were employed to select nonopioid drugs for this review. These included acetaminophen, dexmedetomidine, dexamethasone, ketamine, gabapentin, ibuprofen, ketorolac, diclofenac, magnesium sulfate, clonidine, intravenous lidocaine, and meloxicam (in order of most to least bibliometric interest). Individual reviews on these agents described how the bibliometric indices characterized a drug. They also addressed the question of whether a nonopioid analgesic produced a marked opioid-sparing effect. Information relative to this question was presented *via* the results of meta-analyses with emphasis on the possible reduction of opioid-related side effects. Overall, nonopioid drugs demonstrating the largest popularity among authors and continuous interest growth in 2018-2022 include acetaminophen, dexmedetomidine, dexamethasone, and ibuprofen. The relevant meta-analyses show that nonopioids, administered as components of multimodal analgesia, provided the opioid-sparing effect; they also show that the most common change in the opioid-related side effects was a lower incidence of postoperative nausea and vomiting.

## 1 INTRODUCTION

The current opioid crisis has profoundly influenced many aspects of medical practice [1]. The most dramatic manifestation of the crisis is the increase in the number of deaths involving opioid overdoses, which was first identified in the early 1990s and later became very prominent [2]. The response to the opioid crisis involved many stakeholders in healthcare – practitioners, hospitals, regulators, and pharmacies. These responses profoundly influenced medical practice not only in the ways intended but also in ways unintended and unexpected [1].

Since the beginning of the opioid crisis, the interest in nonopioid analgesics has steadily increased. The desire to reduce the exposure of patients to opioids as a means to avoid potential drug abuse has been a driving force in the use of nonopioids for the treatment of postoperative pain. The introduction of nonopioids was facilitated by the concept of balanced (multimodal) analgesia [3]. The concept of balanced analgesia was based on the premise that the use of a combination of opioid and nonopioid analgesics would improve analgesia and reduce opioid-related side effects [3, 4].

Academic publications are the most obvious place to look for and detect changes in pharmacotherapy trends, including those in the treatment of postoperative pain. Changes in authors’ choices of drugs as the subjects of their articles represent shifts in their interests or/and standards of care. In fact, such choices reflect the opinion of experts most knowledgeable on a subject and also the opinion of those who review and approve publications – the journals’ editorial boards. The current study aimed to analyze the changes in authors’ study choices of nonopioid drugs used in articles on the treatment of postoperative pain during the opioid crisis.

This study aimed to determine how the opioid crisis changed publication-based academic interest in various nonopioid drugs. In addition, changing interest in nonopioid drugs was compared with the interest in another opioid-sparing pain treatment modality – regional anesthesia and its evolving techniques. Publication-based academic interest in nonopioids used for the treatment of postoperative pain was measured *via* specific bibliometric indices [5-8]. This is the first study that determined how the academic interest in nonopioid agents for the treatment of pain has changed over the 30 years of the opioid epidemic. The results of the study are presented in Tables **1** and **2**. Nonopioid drugs with the highest degree of publication-based academic interest have been selected for short individual reviews. Their mechanisms of action are illustrated in Table **3**. Each review describes how the bibliometric indices characterize a nonopioid. They also address the basic question of multimodal analgesia: whether drug combinations, including a nonopioid analgesic, provided an opioid-sparing effect and, as a result, reduced opioid-related side effects [3].

## 2 BACKGROUND

Bibliometric analysis of academic articles on nonopioid drugs for the treatment of postoperative pain was performed using the PubMed database of the US National Library of Medicine. It comprises more than 36 million citations for biomedical literature that are well classified and, most importantly, uses a controlled vocabulary for article indexing (MeSH (Medical Subject Headings) terms). Various bibliometric indices have been developed mainly on the basis of this database [5-8]. The following indices were used: actual number of articles, Index of Change (IC), and Popularity Index (PI). The IC is the percentage change in the number of articles on a specific drug during a 5-year period compared with the previous 5-year period. The PI is the percentage of articles on a certain drug among all articles on the MeSH term “Pain, Postoperative” over the same 5-year period. It allows the assessment of a drug’s comparative popularity among the authors of articles on postoperative pain. While there is a constant growth in the number of drug-related articles in all medicobiological areas, PI allows us to compare a topic’s relative popularity over time.

Search terms related to nonopioid drugs and techniques used for the treatment of postoperative pain were taken from various review articles on this topic [4, 9-12] and from a manual search using the MeSH term “Pain, Postoperative” (PubMed database). The selection of terms was centered on nonopioid drugs used for systemic analgesia. Terms related to regional analgesia techniques were used only for bibliometric comparisons.

The association of the two terms “opioid-sparing” and “opioid-free” (most common in articles on nonopioid treatments for postoperative pain) was used for topic selection. A topic was included in the analysis if the number of PubMed articles on a topic (*i.e*., drug’s name), including at least one of these two names, was 10 or more during the period 2013-2022. As a result, the following topics were included (alphabetical order): “Adductor canal block”, “Acetaminophen”, “Adrenergic alpha-Agonists”, “Adrenergic beta-Antagonists”, “Amantadine”, “Anesthesia Epidural”, “Anesthesia Spinal”, “Aspirin”, “Brachial plexus block”, “Bupivacaine”, “Clonidine”, “Dexamethasone”, “Dexmedetomidine”, “Diclofenac”, “Droperidol”, “Duloxetine”, “Erector spinae plan block”, “Femoral nerve block”, “Flurbiprofen”, “Gabapentin OR Pregabalin”, “Ibuprofen”, “Intercostal block”, “Intravenous lidocaine”, “Ketamine OR Esketamine”, “Ketorolac”, “Levobupivacaine”, “Lidocaine”, “Local infiltration anesthesia”, “Magnesium Sulfate OR Magnesium”, “Meloxicam”, “Methylprednisolone”, “Naproxen”, “Nerve Block”, “Paravertebral block”, “Parecoxib”, “Quadratus lumborum block”, “Rectus sheath block”, Retrolaminar block”, “Ropivacaine”, “Sciatic Nerve block”, “Serratus plane block”, “Transversus abdominis plane block”, “Triamcinolone” (MeSH terms are in bold fonts with initial caps).

The name of a topic was placed in the search box together with the MeSH term “Pain, Postoperative.” The drug or technique was included in the final list for analysis if the number of related articles during 2018-2022 was =/> 100. The following topics did not “make the cut”(Alphabetical order): “Adductor canal block,” “Adrenergic beta-Antagonists,” “Amantadine,” “Aspirin,” “Droperidol,” “Duloxetine,” “Flurbiprofen,” “Intercostal block,” “Methylprednisolone,” “Naproxen,” “Parecoxib,” “Rectus sheath block,” “Retrolaminar block”, “Sciatic nerve block,” “Serratus plane block,” and “Triamcinolone.”

Since the opioid epidemic has been the main driving force behind the increase in the use of nonopioids for the treatment of postoperative pain, its time period was used for grouping bibliometric indices. According to the US Centers for Disease Control and Prevention, a sustained rise in unintentional opioid mortality was observed after 1992 [13]. Accordingly, one 5-year interval (1988-1992) before 1992 and six after 1992 (1993-1997, 1998-2002, 2003-2007, 2008-2012, 2013-2017, 2018-2022) was chosen. Both IC and PC were calculated for all of the above 5-year time intervals if the number of related articles was =/>20.

The primary conclusion of publication-based academic interest in nonopioid medication was based on two principles. One was the degree of popularity among the authors of articles on the treatment of postoperative pain during the most recent 5-year assessment period – 2018-2022. The other was related to the changes in a drug’s popularity during the opioid crisis. Due to the constant growth in the number of PubMed articles, only the PI index allowed the comparisons of a topic's popularity over seven 5-year time periods, from 1988-1992 to 2018-2022.

## 3 RESULTS

### 3.1 General Comparisons

Table **1** presents nonopioid drugs used for the treatment of postoperative pain. A drug was selected for this table if the total number of related articles during 2018-2022 was =/>100. Twelve nonopioid drugs met the requirement and are presented in accordance with their level of publication-based academic interest. In 2018-2022, the number of articles on these drugs varied from 1,260 (acetaminophen) to 119 (meloxicam), with the index of change (IC) being above 100% for dexmedetomidine, ibuprofen, and intravenous lidocaine, and above 50% for acetaminophen, dexamethasone, and meloxicam. In general, the table indicates that during the opioid crisis, the frequency with which a nonopioid was the topic of an article on postoperative pain increased: the PI index went up with all agents. With the nonopioids used in medicine before the 1980s, the PI profound increases occurred earlier, in 1993-1997 or 1998-2002; with the nonopioids introduced in medicine later (such as gabapentin, dexmedetomidine, and meloxicam), the maximum PI was reached later, in 2013-2017 or 2018-2022. With ibuprofen, the increase in PI continued even during the most recent 5-year period, despite the maximum reached in 1998-2002: the ibuprofen PI in 2018-2022 was higher than that in 2013-2017.

Table **2** presents seven regional analgesia techniques used for the treatment of postoperative pain. In 2018-2022, the number of articles on these techniques varied from 611 (erector spinae plane block) to 173 (local infiltration analgesia), with mostly high indexes of change (IC), above 100% for four of the seven types of blocks. The PI index in 2018-2022 was almost always higher than that in 2013-2017. In general, the popularity of these techniques among authors during the opioid crisis increased later than that with nonopioid drugs, and the total number of articles on these techniques was less than that on opioid drugs.

### 3.2 Individual Drug Reviews

#### 3.2.1 Acetaminophen

Although acetaminophen is usually described as a Nonsteroidal Anti-Inflammatory Drug (NSAID), it is largely devoid of anti-inflammatory activity and is regarded as an antipyretic and analgesic [14]. Good efficacy in mild-to-moderate pain and a remarkable safety record resulted in the widespread use of acetaminophen for the treatment of perioperative pain.

When acetaminophen was assessed as a nonopioid for postoperative pain based on the frequency of author choices (Table **1**), it took first place. In 2018-2022, the number of articles on acetaminophen reached 1,260. The IC shows that in 2018-2022, as compared with the previous 5-year period, the number of articles increased by 50%. As far as the PI is concerned, the table indicates that during the six 5-year periods covering the time of the opioid crisis, the popularity of acetaminophen among authors of articles on the treatment of postoperative pain increased. The increase reached its maximum in 1998-2002 -- PI 3.04, compared to 2.08 in the pre-crisis period of 1988-1992. Over the next four 5-year periods, from 2003—2007 to 2018-2022, the PI stayed relatively constant.

The use of nonopioids in postoperative pain was pursuant to the goal of opioid-sparing. Acetaminophen, even if given alone, without other nonopioids, can provide such an effect. The reduction in opioid consumption was usually described as small but statistically significant. Most reliable in this regard are studies involving meta-analysis [15-20]. Some used a Network Meta-Analysis (NMA) [17, 20], which integrates data from direct and indirect comparisons between various nonopioids and a placebo. One of the studies reported a decreased incidence of Postoperative Nausea and Vomiting (PONV) associated with the use of acetaminophen [16].

#### 3.2.2 Dexmedetomidine

Dexmedetomidine has a number of pharmacologic characteristics that make it suitable for use in multimodal analgesic combinations aimed at reducing postoperative opioid consumption. First, dexmedetomidine is a highly selective alpha-2 adrenergic agonist providing sedation and analgesia due to its supraspinal and spinal action [21, 22]. In addition, dexmedetomidine can enhance postoperative analgesia produced by nerve blocks [23, 24]. This effect is peripheral (perineural administration) and not related to its action on alpha-2 adrenoreceptors. The increase in the duration of local anesthetic blocks achieved by adding dexmedetomidine to local anesthetics is produced by blockade of the hyperpolarization-activated cation current [25].

Dexmedetomidine was the second most popular nonopioid analgesic listed in Table **1**. In 2018-2022, the number of articles related to its use for the treatment of postoperative pain reached 840, and its IC was >100%. The PI of dexmedetomidine began to rise only in 2003-2007, later than with many other nonopioid analgesics, due to the date of its initial US Food and Drug Administration (FDA) approval in 1999. However, during the next three time periods (2008-2012, 2013-2017, and 2018-2022), its popularity increased very markedly, more than with any other drug listed in Table **1**. For example, in 2018-2022, the PI of dexmedetomidine rose to 1.94 from 1.37 in 2013-2017.

Dexmedetomidine can reduce postoperative opioid consumption by acting *via* the two mechanisms indicated above. The marked opioid-sparing effect of systemic dexmedetomidine (acting through the alpha-2 adrenergic receptors) was reported in several meta-analyses [17, 26, 27]. The degree of this effect was greater than that reported with acetaminophen [26]. The perineural administration of dexmedetomidine (as a peripheral nerve block adjunct) prolongs the duration of analgesia and reduces postoperative opioid consumption. A meta-analysis of studies on the use of dexmedetomidine to enhance the quality of brachial plexus nerve blocks showed a significant reduction in postoperative morphine consumption [24]. The decrease in opioid consumption induced by dexmedetomidine could be a decisive factor in the reduction of the incidence of PONV [27]. However, the mechanisms underlying PONV are numerous [28], and the effect of dexmedetomidine on nausea and vomiting might be multifactorial. The beneficial analgesic effects of dexmedetomidine should be balanced against an increased risk of hypotension and bradycardia [26].

#### 3.2.3 Dexamethasone

Dexamethasone, a high-potency, long-acting glucocorticoid, has been used to reduce inflammation in a variety of conditions, including inflammation after surgery [29]. It is commonly used to prevent PONV [30]. The systemic analgesic effect of dexamethasone is often explained by its anti-inflammatory action [29]. Dexamethasone has also been shown to prolong peripheral nerve blocks when added to local anesthetics [31].

Dexamethasone is one of the most popular nonopioid agents listed in Table **1**, with 617 articles in 2018-2022. The table’s IC index shows that, compared with the previous 5-year period, in 2018-2022, the number of articles increased by 75%. The IP index of dexamethasone shows a steady increase in every one of the six 5-year periods, including an increase in 2018-2022 to 1.42. This indicates continuing growth in the popularity of dexamethasone among the authors of articles on the treatment of postoperative pain.

The impact of dexamethasone on postoperative analgesia and opioid consumption has been subjected to meta-analysis in a number of studies [17, 32-34]. Most of them concluded that systemic dexamethasone is an effective adjunct in perioperative multimodal strategies: single-dose dexamethasone was associated with small but statistically significant reductions in postoperative opioid consumption. However, significant caution should be taken when routinely including dexamethasone in standard perioperative protocols due to the increased risk of delirium and hypothalamo-pituitary-adrenal axis suppression, especially in elderly patients [35, 36].

#### 3.2.4 Ketamine

Ketamine is available as a racemic mixture and as the S(+) enantomer – esketamine, which is twice as potent as racemic ketamine. The most important pharmacological actions of ketamine are its noncompetitive N-Methyl-D-Aspartate (NMDA) receptor antagonist properties. The anesthetic and analgesic effects of ketamine have been in use for a long time, but the administration of low-dose ketamine for the reduction of postoperative pain is a relatively new development [37, 38]. The mind-altering effect of ketamine and its status as a drug of addiction may play a role in its use [38].

Table **1**, in which ketamine is presented according to the frequency of author choices, shows that the number of articles in 2018-2022 was 502, with an IC index of 34%. The ketamine PI indicates an early rise, reaching a maximum of 1.34 in 1998-2002. During the four subsequent time intervals, its value did not increase.

There are several meta-analyses showing that intravenous ketamine is an effective adjunct for postoperative analgesia [20, 39-41]. In one of these studies [40], ketamine, when added to morphine (or hydromorphone), patient-controlled analgesia, reduced opioid consumption, and postoperative pain intensity. When a network meta-analysis was used to determine the effect of postoperative ketamine on opioid consumption, the decline in consumption was characterized as moderate, but only when used as a postoperative infusion [40]. It was found that when ketamine provided a marked analgesic effect, PONV was less frequent [38-40]. It seems likely that the reduction of PONV with ketamine may be related to decreased opioid consumption.

#### 3.2.5 Gabapentinoids

Gabapentinoids, such as gabapentin and pregabalin, are anticonvulsant agents with analgesic properties. Pregabalin is several times more potent than gabapentin. Both are approved to treat chronic neuropathic pain and are now used perioperatively for the management of postoperative pain.

Table **1** shows that gabapentin and pregabalin (counted together) have 408 articles related to the use of both agents for the treatment of postoperative pain published in 2018-2022. Their combined PI gradually increased from 1998 to 2002 and reached the level of 1.22 from 2013 to 2017.

There are multiple meta-analyses demonstrating that gabapentin [20, 42-45] and pregabalin [20, 44-47] have an opioid-sparing effect when used with opioids for the treatment of postoperative pain. Three of these studies [20, 45, 47] used a network meta-analysis that integrates data from direct and indirect comparisons between various nonopioids and a placebo. One of them [20] reported that the analgesic effects of gabapentinoids, when used in high doses (*i.e*., pregabalin =/>300 mg/day, gabapentin =/>900 mg/day) were moderate in a single-drug regiment (not combined with other nonopioids). One of the studies [44] concluded that gabapentinoids did not produce a significant decrease in opioid consumption or a clinically significant decrease in postoperative pain intensity. While the use of gabapentinoids was associated with a lower risk of PONV, it was also associated with an increase in perioperative sedation and a higher incidence of dizziness. The sedation (and anxiolysis) may have contributed to the decrease in pain score values [40, 44].

#### 3.2.6 Ibuprofen

Ibuprofen is an NSAID that produces analgesic, antipyretic, and anti-inflammatory effects by non-selectively inhibiting cyclooxygenase enzymes. It has been a mainstay in the oral analgesic armamentarium with decades of extensive clinical use and has been released recently as an IV use formulation in many countries.

Ibuprofen is listed in Table **1** as an agent with 409 (2018-2022) articles on the treatment of postoperative pain. This is the highest number of articles among all the table’s NSAIDs (ketorolac, diclofenac, and meloxicam). It also has a high IC value –100%, showing that the number of such articles in 2018-2022 profoundly increased compared with that in the previous 5-year period. At the same time, the PI during all seven 5-year study periods changed little, except for a rise in 2018-2022.

Most NSAID meta-analyses include combined effects of different drugs from the same group. However, several meta-analyses addressed the preoperative administration of intravenous ibuprofen individually [45, 48]. These studies concluded that ibuprofen reduced both postoperative opioid consumption and pain intensity. One of these studies also reported a reduction in PONV [45].

#### 3.2.7 Ketorolac and Diclofenac

These NSAIDs came into medical use in the US in 1989 and 1988, respectively, and have been used for the treatment of postoperative pain.

Table **1** demonstrates that in 2018-2022, the number of articles on ketorolac and postoperative pain was not much different from those on ibuprofen – 384, and the number of diclofenac articles was lower – 185.

The ICs for both drugs show that in 2018-2022, the number of articles (compared with 2013-2017) on ketorolac increased (+35%), but those on diclofenac decreased (23%). As far as the PI is concerned, in 1993-1997, there was a significant increase, up to 2.82 with ketorolac and up to 1.60 with diclofenac. However, a subsequent slow decline of the drug indices over five study periods led to very low levels in 2018-2022: 0.89 with ketorolac and 0.43 with diclofenac. The initial rise of the PI with both drugs in 1993-1997 coincides with the beginning of the opioid crisis; however, the rise may also be a result of their introduction as new agents in the early 1990s.

Meta-analyses of the effects of both agents on postoperative opioid consumption are usually represented in the form of a combined effect of several NSAIDs without an individual drug assessment. One study with individual meta-analysis of ketorolac reported that the drug provided an opioid-sparing effect [49]. The authors concluded that a single dose of ketorolac reduced postoperative pain and PONV.

#### 3.2.8 Magnesium Sulfate

Magnesium sulfate may have a role as an analgesic adjuvant for a variety of acute and chronic pain. Though its analgesic properties are most commonly associated with its action as a noncompetitive NMDA receptor antagonist, it also acts as a physiologic Ca^2+^ antagonist [50]. Magnesium sulfate can be administered intravenously to augment postoperative opioid analgesia. It is also used as an additive to local anesthetics to prolong peripheral nerve blocks, however, perineural application has not gained wide use [51].

Table **1**, in which magnesium sulfate is presented according to the frequency of authors’ choices, demonstratesthat the number of articles in 2018-2022 was 182 with an IC index of 28%. The PI for magnesium sulfate began to rise only after 2003-2007, and its rather low maximum of 0.48 was reached in 2013-2017.

The impact of systemic magnesium sulfate on postoperative opioid consumption was reviewed in several meta-analyses [52-55]. They showed that magnesium sulfate reduced postoperative opioid consumption; the reduction in postoperative pain scores was smaller. A network meta-analysis of the effect of magnesium sulfate, added to local anesthetics for upper-extremity regional anesthesia, showed that it prolonged the duration of analgesia [56].

#### 3.2.9 Last Qualifying Agents

The last three agents presented in Table **1** (clonidine, intravenous lidocaine, and meloxicam) have many fewer 2018-2022 articles than the table’s other drugs. Clonidine is a selective (alpha-2: alpha-1 = 20: 1) adrenergic receptor agonist. However, its selectivity is almost ten times lower than that of dexmedetomidine, which may have been one of the factors leading to a decline in its use for the treatment of postoperative pain. Although the number of 2018-2022 articles on intravenous lidocaine placed it at the bottom of Table **1**, its IC index is rather high at >100%. Meloxicam is an NSAID with some selectivity in the inhibition of COX-2 to COX-1 enzymes (cyclooxygenase), especially at lower doses, though parecoxib and celecoxib are much more selective COX-2 inhibitors [14]. The introduction of an intravenous formulation of meloxicam supported its use in the treatment of postoperative pain. Both meloxicam and intravenous lidocaine have rather low PI indices.

## 4 DISCUSSION

The desire to reduce the exposure of patients to opioids remains a driving force in the use of nonopioid drugs for the treatment of postoperative pain. There is no reliable evidence that reduction of immediate postoperative opioid exposure has any effect on the likelihood of drug abuse; at the same time, the introduction of nonopioids for the treatment of postoperative pain may be an important factor in another respect. Postoperative pain remains poorly treated, and there has been little overall progress in this regard in the last 20 years [1, 57, 58]. Combined administration of opioids with nonopioids (including regional analgesia techniques) may be a means to improve pain treatment.

Nonopioid drugs most frequently chosen by the authors of articles on the treatment of postoperative pain include acetaminophen, dexmedetomidine, dexamethasone, gabapentin (including pregabalin), ketamine (esketamine), ibuprofen, ketorolac, diclofenac, magnesium sulfate, clonidine, intravenous lidocaine, and meloxicam (from most to least interest). They are used to provide opioid-sparing effects in the framework of balanced (multimodal) analgesia. Because the individual contribution of each drug in an analgesic combination can be rather small, the assessment of the magnitude of such individual effects is difficult. The most valid approach in this regard is meta-analysis. Systematic review and meta-analysis can determine the difference in analgesic efficacy between two interventions, but they are limited in evaluation of evidence due to the high variability of many parameters in postoperative pain management among included studies, which often yields insufficient data. The relatively new method of meta-analysis called – network meta-analysis [59] – increases the amount of data on drug efficacy by integrating data not only from direct but also from indirect comparisons between all available analgesic comparisons. As indicated in the above drug reviews, the related meta-analyses (including network meta-analyses) show that nonopioids, administered as components of multimodal analgesia, provided an opioid-sparing effect, which was individually confirmed for almost all of the reviewed nonopioids.

In accordance with the concept of balanced analgesia, [3, 4] opioid-sparing would reduce opioid-related side effects, such as nausea, vomiting, constipation, sedation, and ventilatory depression. The findings of the above meta-analyses regarding opioid side effects related to the use of nonopioid drugs show that the most common change in those side effects was a lower incidence of postoperative nausea and vomiting (PONV). This change was reported with acetaminophen, dexmedetomidine, dexamethasone, ketamine, gabapentin, and ibuprofen, though it is worth mentioning that the lower risk of PONV with dexmedetomidine and dexamethasone might be multifactorial. Different nonopioid drugs have different ranges of side effects depending on their doses and specific combinations with other agents administered in the perioperative period. As a result, the use of various drug combinations without a proper understanding of their interactions can be problematic [60]. It should be added here that the short-term use of NSAIDs (including ketorolac) for postoperative analgesia is not statistically associated with increased postsurgical bleeding risk [61].

The assessment of the opioid-sparing effect of nonopioids is subject to the usual problem associated with analgesia measurement: any reduction in postoperative opioid consumption is often obscured by the interaction between two main analgesia outcome measures – opioid consumption and pain intensity. Absent a difference in pain scores between the drug group and placebo group, opioid consumption becomes the sole measure of the analgesic effect of a drug. However, it is very common to have a difference between drug and placebo groups in both outcome measures. As a result, changes in one outcome (pain score) may counterbalance changes in another outcome (opioid consumption), resulting in a failure to reach statistical significance for one or both outcomes. There are several reasons for this problem, [62] the most prominent of which is the weak correlation between pain intensity and opioid consumption [63]. Ketamine is the only drug listed in Table **1** that, in a large dose, has the capacity to provide anesthesia and profound analgesia; however, for postoperative analgesia, it is used in low doses. Ketamine is an NMDA receptor antagonist and can attenuate the development of acute tolerance to opioid analgesia. This effect provides an alternative explanation for the reduction in opioid consumption seen with ketamine-opioid postoperative analgesia: not only the effect of ketamine-induced analgesia but also the restoration of the full effect of opioid analgesia [64].

In addition to nonopioid drugs, opioid-sparing strategies also include various regional analgesia techniques [65-68]. Table **2** shows that the popularity of these techniques among the authors of articles on the treatment of postoperative pain is overall high but lower than that with nonopioid drugs. This may be due to the fact that the use of regional analgesia techniques as opioid-sparing strategies in the opioid crisis accelerated later than that of the nonopioid drugs. That relative delay might have been due to a number of limitations with the use of these techniques. For example, traditional local anesthetic-based nerve blocks can produce motor weakness, decreasing the ability of patients to participate in postoperative rehabilitation. More importantly, limited-duration regional analgesia action might require the use of continuous local anesthetic blockade with the risk of catheter-associated infection and dislodgments. Moreover, continuous peripheral nerve blocks were technologically difficult at the start of the opioid epidemic, and suitable equipment became readily available only during later decades. Ultimately, ultrasound guidance was introduced and widely adopted in regional anesthesia after 2005 allowing for a significant increase in access to regional anesthesia, Including continuous blocks, and for the design and introduction of many new block techniques. Many of these new techniques target fascial planes (*e.g*., erector spinae plane block, transversus abdominis plane block) and the distal nerve branches that do not have significant motor nerve components. Such blocks cause no significant weakness or functional recovery impairment and delay.

Changes in publication-based academic interest in drugs are usually rather slow. For example, the most common outcome of the introduction of a new drug is the decline of a number of articles on an old drug that was previously dominant for the same purpose (like sumatriptan *vs*. ergotamine in migraine). However, the expected decline is extremely slow: it takes 15-20 years to supplant interest in an old drug with a new one by 50-70% [69]. This may suggest that the levels of publication-based interest in nonopioids observed in 2018-2022 may continue for a long time.

## CONCLUSION

This review examines the changes in collective subject choices made by authors of articles on nonopioids used for the treatment of postoperative pain. It covers 12 drugs over seven 5-year periods, the last of which is 2018-2022. Overall, nonopioid drugs demonstrating the largest popularity among authors and continuous interest growth in 2018-2022 include acetaminophen, dexmedetomidine, dexamethasone, and ibuprofen (Table **4**). The relevant meta-analyses show that nonopioids, administered as components of multimodal analgesia, provided opioid-sparing effects; they also show that the most common change in the opioid-related side effects was a lower incidence of postoperative nausea and vomiting.

## Figures and Tables

**Table 1:** Publication-based academic interest in nonopioid drugs for the treatment of postoperative pain.

**#**	**Name**	**Number of Articles** **2018-2022**	**IC** **2018-2022**	**PI**						
				**88- 92**	**+ 93-97**	**98-02**	**03-07**	**08-12**	**13-17**	**18-22**
1.	Acetaminophen	1.260	52	2.08	2.62	3.04*	3.02	2.85	2.80	**2.91**
2.	Dexmedetomidine	840	>100	-------	-------	-------	0.28	0.52	1.37	**1.94**
3.	Dexamethasone	617	74	-------	0.33	0.46	0.68	0.93	1.20	**1.42**
4.	Gabapentin, Pregabalin	480	33	-------	-------	0.21	0.71	1.04	1.22*	1.11
5.	Ketamine, Esketamine	502	34	0.39	0.68	1.34*	1.29	1.29	1.27	1.16
6.	Ibuprofen	409	>100	0.87	0.79	1.02*	0.85	0.72	0.68	**0.91**
7.	Ketorolac	384	35	1.09	2.82*	1.69	1.12	1.02	0.96	0.89
8.	Diclofenac	185	-23	1.03	1.60*	1.59	1.31	1.02	0.81	0.43
9.	Magnesium Sulfate	182	28	-------	-------	-------	0.15	0.36	0.48*	0.42
10.	Clonidine	129	-15	0.86	1.24*	1.17	1.07	0.61	0.51	0.30
11.	Intravenous Lidocaine	127	>100	-------	-------	-------	-------	0.15	0.20	**0.29**
12.	Meloxicam	119	80	-------	-------	-------	0.20	0.21	0.22	**0.28**

**Note:** IC – Index of change, the percentage change in the number of articles on a specific drug during 2018-2022 period compared with the previous 5-year period (2013-2017). PI – Popularity index, popularity of a drug among the authors of the articles on postoperative pain. It represents the percentage of articles on a drug among all articles in the related area published over the same time period. +Initial sustained rise of unintentional opioid mortality. *Previous maximum (if above 2018-2022 value). In bold -- if above 2013-2017 value.

**Table 2:** Publication-based academic interest in various regional analgesia techniques for the treatment of postoperative pain.

**#**	**Name**	**Number of Articles** **2018-2022**	**IC** **2018-2022**	**PI**						
				**88-92**	**+ 93-97**	**98-02**	**03-07**	**08-12**	**13-17**	**18-22**
1	Erector spinae plane block	611	>100	-------	-------	-------	------	-------	0.04	**1.41**
2	TAP block	394	65	-------	-------	-------	-------	0.38	0.80	**0.91**
3	Paravertebral block	344	>100	-------	-------	-------	0.24	0.43	0.59	0.79
4	Brachial plexus block	285	>100	-------	0.27	0.40	0.41	0.40	0.46	**0.66**
5	Quadratus lumborum block	280	>100	-------	-------	-------	-------	-------	0.10	**0.65**
6	Femoral nerve block	207	-15	-------	-------	0.25	0.41	0.51	0.82*	0.48
7	Local infiltration analgesia	173	57	-------	-------	-------	-------	0.18	0.37	**0.40**

**Note:** IC – Index of change, the percentage change in the number of articles on a specific drug during 2018-2022 period compared with the previous 5-year period (2013-2017). PI – Popularity index, popularity of a drug among the authors of articles on postoperative pain. It represents the percentage of articles on a drug among all articles in the related area published over the same time period. + Initial sustained rise of unintentional opioid mortality. TAP—Transversus Abdominis Plane Block *Previous maximum (if above 2018-2022 value). In bold -- if above 2013-2017 value.

**Table 3:** Basic nonopioid drugs and mechanisms of their actions.

**Drug Class**	**Mechanism of Action**	**Popular Drugs**
NSAIDs	Inhibit the COX enzymes and PG production	Acetaminophen, ibuprofen, ketorolac, diclofenac
Alpha-2 Adrenergic Receptor Agonists	Activate the alpha-2 receptors	Dexmedetomidine
Glucocorticoids	Modulate the expression of corticosteroid-responsive genes	Dexamethasone
N-Methyl-D-Aspartate Receptor Antagonists	Inhibit the NMDA receptors	Ketamine
Anticonvulsants	Block the Na^+^ and Ca^2+^ channels	Gabapentin

**Abbreviations:** NSAIDs – nonsteroidal anti-inflammatory drugs, COX – cyclooxygenase, PG – prostaglandin, NMDA – N-Methyl-D-Aspartate.

**Table 4:** Nonopioids for the treatment of postoperative pain.

Drugs used during	Acetaminophen, Dexmedetomidine,
opioid crisis,	Dexamethasone, Ketamine, Gabapentin,
1993-2022	Ibuprofen, Ketorolac, Diclofenac,
-	Magnesium sulfate, Clonidine,
-	Intravenous lidocaine, and Meloxicam
The most popular*	**Acetaminophen, Dexmedetomidine,**
in 2018-2022	**Dexamethasone, and Ibuprofen**

**Note:** *among the authors of academic articles.

## LIST OF ABBREVIATIONS

FDA: US Food and Drug Administration
IC: Index of Change
MeSH: Medical Subject Headings Terms
NMA: Network Meta-Analysis
NMDA: N-Methyl-D-Aspartate
NSAID: Nonsteroidal Anti-inflammatory Drug
PI: Popularity Index
PONV: Postoperative Nausea and Vomiting
